# Genetic diversity and population structure analysis of *Lateolabrax maculatus* from Chinese coastal waters using polymorphic microsatellite markers

**DOI:** 10.1038/s41598-021-93000-6

**Published:** 2021-07-27

**Authors:** Wei Wang, Chunyan Ma, Longling Ouyang, Wei Chen, Ming Zhao, Fengying Zhang, Yin Fu, Keji Jiang, Zhiqiang Liu, Heng Zhang, Lingbo Ma

**Affiliations:** 1 Key Lab of Marine and Estuarine Fisheries Resources and Ecology, Ministry of Agriculture, East China Sea Fisheries Research Institute, Chinese Academy of Fishery Sciences, No. 300 Jungong Road, Yangpu District, Shanghai, 200090 China; 2 East China Sea Fisheries Research Institute, Chinese Academy of Fishery Sciences, Shanghai, 200090 China

**Keywords:** Genetic markers, Population genetics, Biodiversity, Population dynamics

## Abstract

In order to provide valuable guidelines for the conservation of germplasm of *Lateolabrax maculatus*, the genetic diversity and population structure analysis were evaluated for eight geographic populations along coastal regions of China, using 11 microsatellite DNA markers. The genetic parameters obtained showed that, eight populations can be clustered into two groups, the Northern group and the Southern group, concordant with their geographical positions. The UPGMA tree constructed according to the Nei’s genetic distance along with the structure analysis and discriminant analysis of principal component also supported this result. This might be explained by the geographic separation and the divergent environmental conditions among the populations. It's worth noting that, QD (Qingdao) population from northern area was assigned to the Southern group and showed a close genetic relationship and similar genetic constitution with the southern populations. We speculated that large scales of anthropogenic transportation of wild fries from QD populations to the southern aquaculture areas in history should be the primary cause. The populations from GY (Ganyu), RD (Rudong) and BH (Binhai) had higher genetic diversity and showed limited genetic exchange with other populations, indicating better conservation of the natural resources in these regions. All populations were indicated to have experienced bottleneck events in history.

## Introduction

Chinese sea bass, *Lateolabrax maculatus*, is an economically important species for Asian countries. In China, it is a primary aquaculture marine fish widely distributed along the coastal areas. However, the long-term over-fishing of both fry and adult fish, habitat destruction, water pollution, and other factors resulted in dramatic decline of their wild resources. The wild fries were almost extinct around the Yangtze estuary^1^. Moreover, introgression of the breeding offspring and adult fish escaped from culture ponds or cages also negatively impacted on the biodiversity of the wild resources of *L. maculatus*, similar to what has been proved in *Salmo salar*^2,3^. Therefore, there was an urgent need to protect the *L. maculatus* wild resources in China with scientific approaches.

It is widely accepted that the knowledge of the genetic studies of wild stocks can be performed using molecular markers such as mtDNA, microsatellite loci and isoenzyme. Among these, microsatellites have provided very useful data comparing the genetic diversity between wild and cultured populations of marine species, aiding in the conservation management of over-exploited populations and the corresponding policy design^4–6^. Although several genetic studies of this species had been reported in China, they were mainly focused on *L. maculatus* populations in partial areas, such as Shandong Peninsula, and the sample sizes analyzed were relatively small. Besides, most of these studies were outdated (before 2010)^1,7–13^. The genetic diversity and population structure of *L. maculatus* might have changed over the past ten years. One recent study in 2017 showed, a lower nucleotide diversity and a higher haplotype diversity indicating *L. maculatus* in China has experienced potential population expansion. However, a significant divergence was only found between Qingdao and Fangcheng populations based on mitochondrial COI gene sequences from all five populations analyzed^14^. Another study of six geographic populations of *L. maculatus* suggested that this species could be divided into two groups, i.e. Southern and Northern group according their geographic locations^15^. Apparently, these results are insufficient to provide a comprehensive understanding of current genetic background of wild *L. maculatus* resources in China. There is renewed interest in updating the genetic information of this species.

In our study, the genetic diversity within and among the eight wild *L. maculatus* geographic populations collected from the coastal regions in China and their population structures were investigated using 11 microsatellite DNA markers. It is expected that this study can largely enrich the genetic information of *L. maculatus* flocks in China, which can be used for not only the protection of its wild resources, but also for improving the developing sustainable fishing management policy.

## Results

### Genetic diversity

The genetic diversity indices of 11 microsatellite loci in eight *L. maculatus* populations are shown in Table 1. In total, 316 alleles were detected in 294 individuals, with an average value of 28.7273 alleles per loci. The highest Ne (expected number of alleles) (19.7277) value was found in Lama 31 locus, while the Lama 28 locus exhibited the lowest number of Ne (1.861). The PIC (polymorphic information content) values lay in the range from 0.4328 (Lama 28) to 0.9470 (Lama 31) with a mean value of 0.8092. All loci showed a high polymorphic (PIC > 0.5) except Lama 28 with a medium value (0.5 > PIC > 0.25). The Fis (inbreeding coefficient) value per locus averaged 0.1325, ranging from 0.0009 (Lama 21) to 0.4368 (Lama 10), while the Fit (total in-coefficient of population) value was 0.2362 averagely. Both MICROCHECKER and FreeNA revealed evidence for presence of null alleles in all loci. However, as null allele frequencies for each locus computed by EM algorithm were all much less than 0.2 (ranged from 0.0131 to 0.1135) (Table 1), and each *L. maculatus* populations analyzed in present study consisted of at least 25 individuals, the existence of null alleles was considered not to affect the results of following genetic analysis^6,16,17^.

**Table 1:** Genetic diversity of 11 microsatellite loci in eight *L. maculatus* populations.

Locus	Na	Ne	Ho	He	PIC	I	HWE	P_N_	Fis	Fit	Fst
Lama 04	23.0000	4.2220	0.5238	0.7644	0.7417	1.9064	0.0000	0.0694	0.1966	0.3088	0.1396
Lama 10	23.0000	5.8124	0.4302	0.8295	0.8130	2.2182	0.0000	0.1965	0.4368	0.4870	0.0892
Lama 21	29.0000	10.5124	0.8299	0.9068	0.8979	2.6765	0.9841	0.0131	0.0009	0.1001	0.0993
Lama 23	32.0000	7.8937	0.7979	0.8748	0.8606	2.4129	0.9999	0.0231	0.0295	0.0987	0.0713
Lama 24	40.0000	6.5374	0.6860	0.8485	0.8351	2.4619	1.0000	0.0364	0.0611	0.1967	0.1445
Lama 28	8.0000	1.8610	0.3049	0.4637	0.4328	0.9473	0.0000	0.1135	0.3571	0.6683	0.4841
Lama 31	44.0000	19.7277	0.8900	0.9509	0.9470	3.2455	1.0000	0.0123	0.0354	0.0673	0.0331
Lama 32	25.0000	5.5269	0.5461	0.8205	0.8048	2.2308	0.0000	0.0727	0.2127	0.3355	0.1560
Lama 36	17.0000	6.278	0.7082	0.8422	0.8219	2.0793	0.0000	0.0437	0.0983	0.1728	0.0826
Lama 38	23.0000	5.864	0.5119	0.8309	0.8104	2.1341	0.0000	0.0957	0.2472	0.3795	0.1757
Lama 44	52.0000	16.4509	0.8908	0.9408	0.9360	3.2095	1.0000	0.0136	0.0213	0.0525	0.0318
Mean	28.7273	8.2442	0.6473	0.8248	0.8092	2.3202		0.0627	0.1325	0.2362	0.1196

*Na* observed number of alleles, *Ne* expected number of alleles, *Ho* observed heterozygosity, *He* expected heterozygosity, *PIC* polymorphic information content,* I* shannon wiener index, *HWE* deviations from Hardy–Weinberg equilibrium, *P*_*N*_ frequency of null alleles, *Fis* inbreeding coefficient, *Fit* total in-coefficient of population.

The genetic diversity parameters of eight *L. maculatus* populations based on 11 microsatellite markers are listed in Table 2. The results indicated that GY population had the highest genetic diversity among eight populations, while the lowest genetic parameter values were mostly observed in LY population. In general, GY, BH and RD (Rudong population) had the higher genetic diversity comparing to other populations.

**Table 2:** Genetic diversity statistics of eight *L. maculatus* populations.

	Na	Ne	I	Ho	He	PIC
QD	10.0000	4.8794	1.6515	0.6289	0.7068	0.6742
FC	10.5455	5.6762	1.7896	0.6470	0.7580	0.7219
DT	8.5455	4.5958	1.6216	0.6131	0.7270	0.6822
LY	9.4545	4.4636	1.5253	0.6441	0.6640	0.6273
CM	10.0909	4.7693	1.6582	0.6133	0.7115	0.6782
GY	18.0000	10.3263	2.4847	0.7728	0.9120	0.8730
RD	11.6364	6.1103	1.8850	0.6016	0.7896	0.7501
BH	15.3636	8.3063	2.2311	0.6813	0.8573	0.7153

*LY* Lieyu, *CM* Chongming, *DT* Dongtou, *QD* Qingdao, *FC* Fangcheng, *GY* Ganyu, *RD* Rudong, *BH* Binhai, *Na* observed number of alleles, *Ne* expected number of alleles; *I* shannon wiener index, *Ho* observed heterozygosity, *He* expected heterozygosity, *PIC* polymorphic information content.

### Genetic differentiation and population structure

The pair-wise Fst (genetic differentiation coefficient values) and Nm (gene flow) value among eight *L. maculatus* populations are shown in Table 3. The Fst values between each two populations ranged from 0.0110to 0.1852, and the genetic differentiation between each two populations reached a significant level (*P* < 0.01). The highest Fst value was observed between RD and LY populations, while the lowest value was found for FC (Fangcheng) and QD (Qingdao) populations. GY, RD and BH populations showed high level differentiation when compared to the other five populations. These results suggested that all populations could be divided into two groups, GY, RD and BH could be classified into one group, while the remaining five populations were assigned to the other group. Consistent with the results of genetic differentiation, eight populations could be clustered into two groups based on pair-wise Nm values. The gene flow between GY, RD, BH and other five populations (Nm < 2.2) were much lower than that between each two of those three populations (Nm > 3.1).

**Table 3:** Genetic differentiation coefficient (Fst, below diagonal), gene flow (Nm, above diagonal) and inbreeding coefficient (Fis, on diagonal) for eight *L. maculatus* populations.

	QD	FC	DT	LY	CM	GY	RD	BH
QD	0.0525	22.4773	7.7245	2.1360	3.5264	1.4995	1.2719	1.7142
FC	0.0110*	0.1416	5.5721	1.9113	2.7884	1.7354	1.3882	1.9315
DT	0.0314*	0.0429*	0.1430	4.1873	13.4262	1.7900	1.4277	2.1109
LY	0.1048*	0.1157*	0.0563*	0.0362	14.5604	1.4565	1.0998	1.6676
CM	0.0662*	0.0823*	0.0183*	0.0169*	0.1000	1.6723	1.3370	1.9186
GY	0.1429*	0.1260*	0.1226*	0.1465*	0.1301*	0.0892	3.1514	9.6471
RD	0.1643*	0.1526*	0.1490*	0.1852*	0.1575*	0.0735*	0.2271	5.0032
BH	0.1273*	0.1146*	0.1059*	0.1304*	0.1153*	0.0253*	0.0476*	0.1955

*LY* Lieyu, *CM* Chongming, *DT* Dongtou, *QD* Qingdao, *FC* Fangcheng, *GY* Ganyu, *RD* Rudong, *BH* Binhai

*P < 0.01.

**Figure 1:**
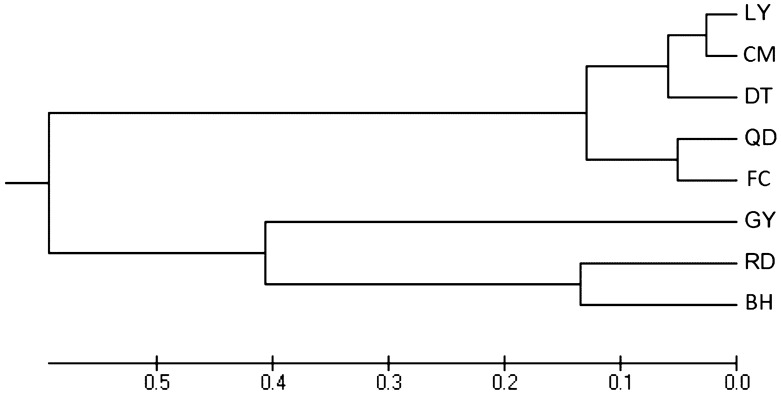
UPGMA clustering tree of *L. maculatus* populations based on Nei's distance. *LY* Lieyu, *CM* Chongming, *DT* Dongtou, *QD* Qingdao, *FC* Fangcheng, *GY* Ganyu, *RD* Rudong, *BH* Binhai.

Consistent with the results of genetic differentiation and gene flow, GY, RD and BH populations showed large genetic distance and low genetic identity when compared to the other five populations (Table 4). As a result, all eight populations are grouped into two main genealogical branches in Fig. 1. RD and BH populations converged first, then gathered with GY population and separated from other populations. The remaining five populations formed the other branch. Among them, QD and FC clustered as a group. In the other group, DT population was separated, while LY and CM populations clustered as a small branch, which represented a closer relationship between the two.

**Table 4:** Nei's genetic identity (above diagonal) and genetic distance (below diagonal) for eight *L. maculatus* populations.

	QD	FC	DT	LY	CM	GY	RD	BH
QD	****	0.9035	0.8835	0.7565	0.8290	0.1968	0.3239	0.2891
FC	0.1015	****	0.8032	0.6572	0.7251	0.1919	0.3028	0.3042
DT	0.1239	0.2191	****	0.8592	0.9195	0.2539	0.3515	0.3280
LY	0.2791	0.4197	0.1518	****	0.9494	0.3289	0.3701	0.3910
CM	0.1876	0.3215	0.0839	0.0519	****	0.2960	0.3816	0.3660
GY	1.6255	1.6506	1.3708	1.1119	1.2173	****	0.4037	0.4881
RD	1.1272	1.1946	1.0455	0.9940	0.9633	0.9070	****	0.7643
BH	1.2409	1.1899	1.1146	0.9391	1.0052	0.7173	0.2689	****

*LY* Lieyu, *CM* Chongming, *DT* Dongtou, *QD* Qingdao, *FC* Fangcheng, *GY* Ganyu, *RD* Rudong, *BH* Binhai.

An analysis of molecular variance (AMOVA) test was performed in order to evaluate the genetic diversity among and within populations. As presented in Table 5, 89.86% of total variations were found among populations, and 10.14% were observed within populations. The fixation index was 0.10140 (Table 5).

**Table 5:** AMOVA results for eight *L. maculatus* populations.

Source of variation	d.f	Sum of squares	Variance components	Percentage of variation	Fst	P
Among populations	7	198.889	0.34648 Va	10.14	0.10140	0.00000
Within populations	580	1780.956	3.07061 Vb	89.86		
Total	587	1979.845	3.4171			

The mutation-drift equilibrium tests were performed for eight *L. maculatus* populations in this study. As shown in Table 6, none of all populations deviate from equilibrium under IAM and TPM models in Sign Test (*P* > 0.05), except for BH. However, all populations showed a high percent of heterozygous deficiency under SMM model, and significantly deviated from mutation-drift equilibrium (*P* < 0.01). This can be supported the positive value of Fis obtained in present study (Table 3). In Wilcoxon Sign-rank Test, BH population showed no deviation from mutation-drift equilibrium under all three models (*P* > 0.05). In contrast, QD, FC, LY and CM populations deviated from equilibrium with extreme significance (*P* < 0.01), while DT, GY and RD population deviated from equilibrium significantly under SMM model (*P* < 0.05). In addition, FC population showed significant deviations under both IAM model (*P* < *0.05*) and SMM model (*P* < 0.01).

**Table 6:** Results of mutation-drift equilibrium tests of eight *L. maculatus* populations.

	Sign test						Wilcoxon Sign-rank test		
	IAM		TPM		SMM		IAM	TPM	SMM
	He/Hd	*P*	He/Hd	*P*	He/Hd	*P*	*P*	*P*	*P*
QD	7/4	0.53554	4/7	0.09504	1/10	0.00104**	0.46484	0.14746	0.00244**
FC	9/2	0.11644	6/5	0.47553	1/10	0.00096**	0.01221*	0.96582	0.00684**
DT	6/5	0.46898	5/6	0.24571	2/9	0.00688*	0.32031	0.27832	0.01611*
LY	7/4	0.50164	6/5	0.47852	1/10	0.00085**	0.70020	0.46484	0.00098**
CM	7/4	0.52851	4/7	0.11366	0/11	0.00005**	0.17480	0.14746	0.00049**
GY	8/2	0.20461	4/6	0.10082	1/9	0.00308**	0.13086	0.32227	0.00488*
RD	9/2	0.11578	7/4	0.50695	1/10	0.00101**	0.12305	0.89844	0.00342**
BH	10/1	0.03228*	7/4	0.53376	2/9	0.00594**	0.05371	1.00000	0.05371

*LY* Lieyu, *CM* Chongming, *DT* Dongtou, *QD* Qingdao, *FC* Fangcheng, *GY* Ganyu, *RD* Rudong, *BH* Binhai, *He/Hd* represents the ratio of heterozygote excess and heterozygous deficiency loci number.

*Means significant deviation from equilibrium (*P* < 0.05).

**Means extremely significant deviation from equilibrium (*P* < 0.01).

**Figure 2:**
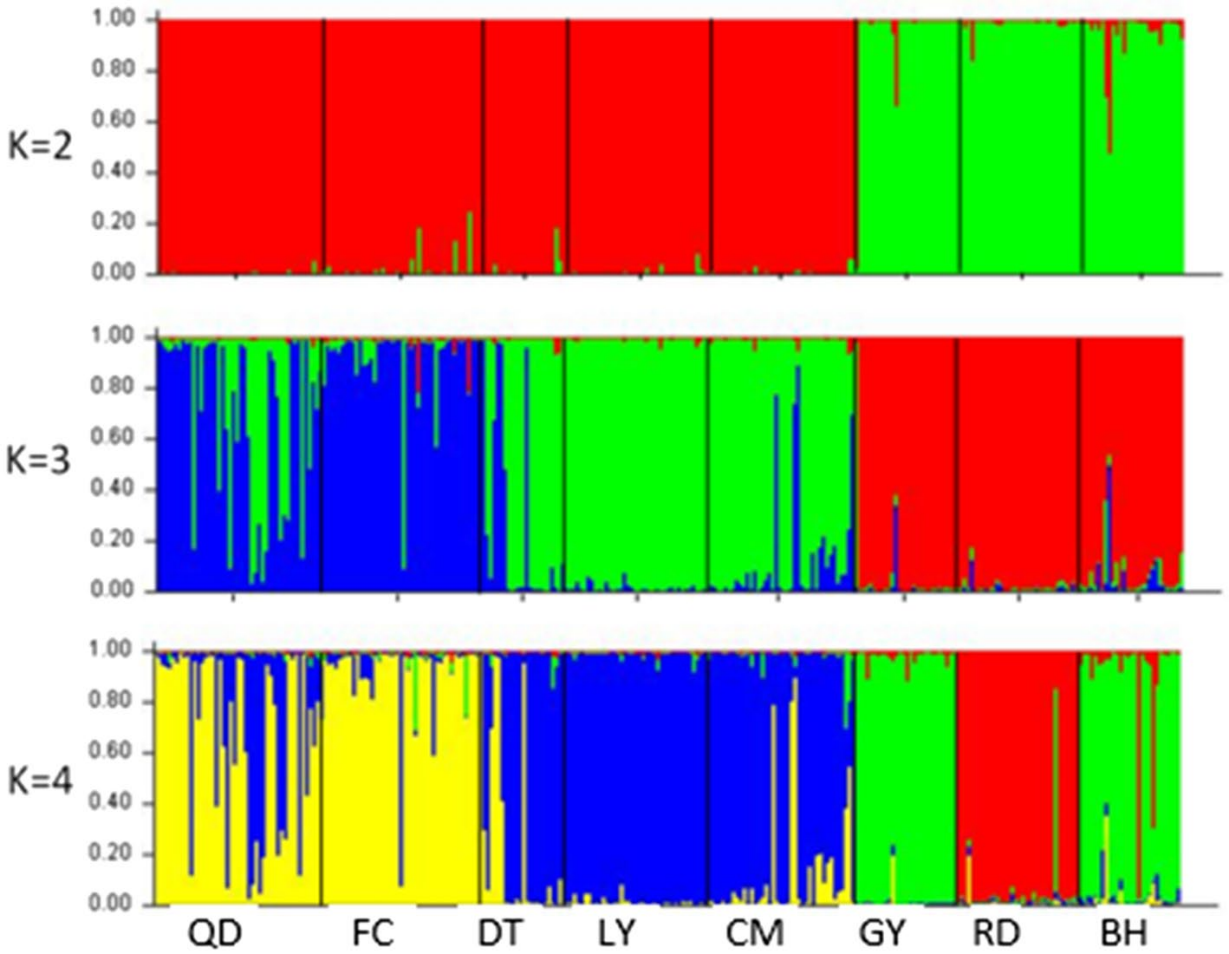
STRUCTURE genetic cluster analysis for *L. maculatus* populations. *LY* Lieyu, *CM* Chongming, *DT* Dongtou, *QD* Qingdao, *FC* Fangcheng, *GY* Ganyu, *RD* Rudong, *BH* Binhai. The population names were given below the box plot with the individuals of different populations separated by vertical black lines. Each color represents “one population”.

**Figure 3:**
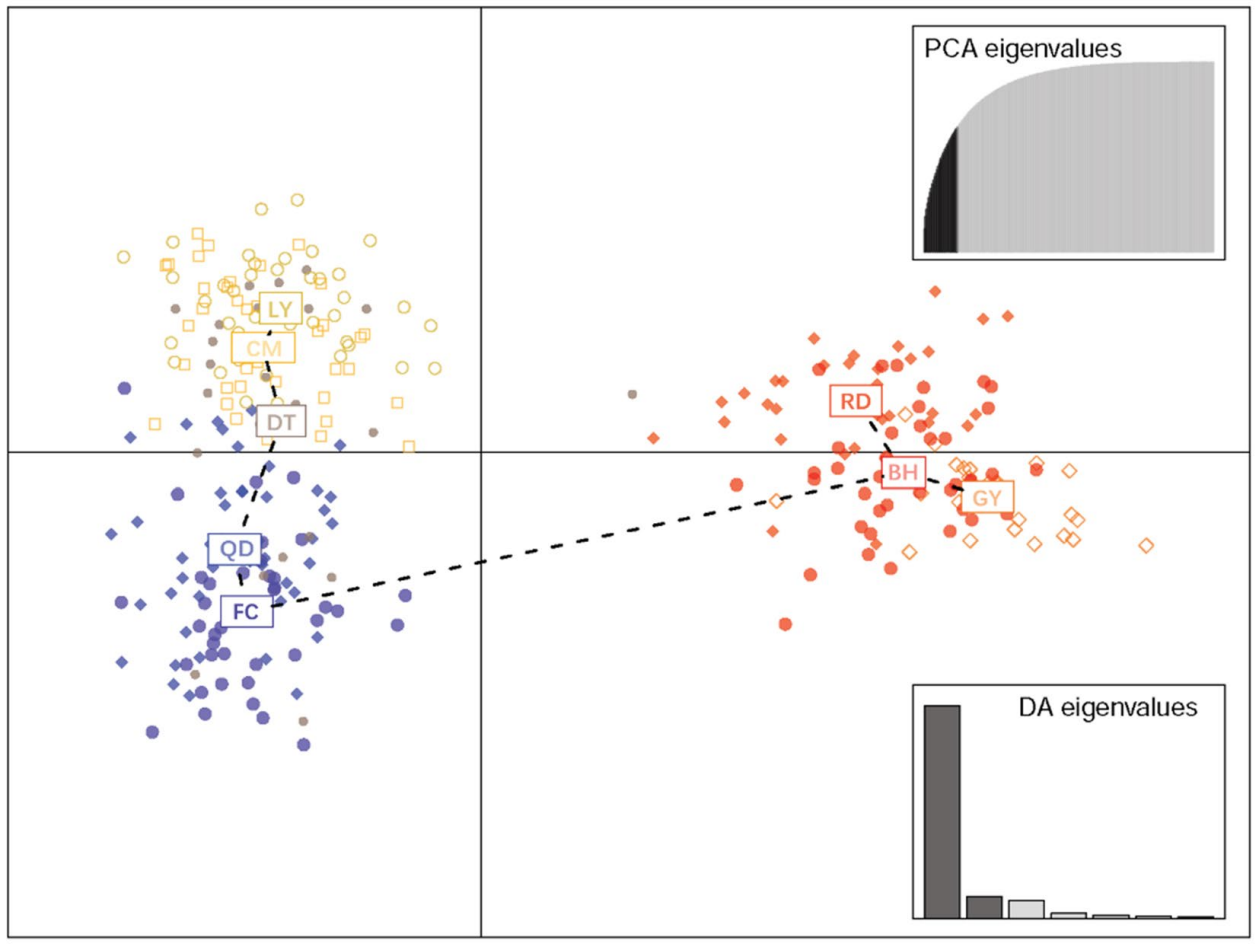
DAPC plot diagram of *L. maculatus* populations. *LY* Lieyu, *CM* Chongming, *DT* Dongtou, *QD* Qingdao, *FC* Fangcheng, *GY* Ganyu, *RD* Rudong, *BH* Binhai. Colored dots with different shapes represent individuals from different geographical populations, PCA and DA scatterplots on the right side of the graph indicate the principal components and numbers of discriminant functions for the computations.

The proportion of membership for *L. maculatus* across eight populations was generated under different cluster numbers (K values) by STRUCTURE software (Fig. 2). When K equaled to 2, most individuals from QD, FC, DT, LY and CM populations were assigned to cluster 1, while most individuals of GY, RD and BH populations were assigned to cluster 2. When K equaled to 3, individuals were divided into three clusters, with most individuals of QD and FC assigned to cluster 1, and part of QD population assigned to cluster 3. Most individuals from DT, LY and CM populations were assigned to cluster 3, while most individuals of GY, RD and BH populations were assigned to cluster 2. When K equaled to 4, most individuals of QD, FC, DT, LY and CM populations showed similar genetic structure to that of K equaled to 3. Most individuals of GY and BH were assigned to cluster 3, meanwhile most individuals from RD population were assigned to cluster 4. A similar genetic structure among the individuals from eight populations was also obtained by DAPC (Discriminant Analysis of Principal Component) (Fig. 3).

## Discussion

### Polymorphism diversity analysis of microsatellite markers

In the study of population genetics, molecular markers such as isozyme, mtDNA and microsatellites DNA have been widely used to monitor the genetic diversity within and between populations of many fishery species, including *L. maculatus*^12,14,18–21^. Compared with the other molecular markers, microsatellites have many advantages, including co-dominant inheritance, highly polymorphism and random dispersion in the genome^22^. However, only a few researches of *L. maculatus* applied microsatellites, although a number of microsatellite markers have been developed^23–25^. In our study, 11 microsatellite loci originally described by Shao et al^23^ were tested. All loci were successfully amplified. The mean value of Na (28.7273) and PIC (0.8092) for them were both higher than that in former studies of *L. maculatus* based on microsatellite^14,23,24^. Also, 10 of the 11 loci verified in this study present a high level of polymorphism diversity (PIC > 0.5)^26^. These results indicated that the microsatellite loci tested in present study were effective molecular genetic markers and could be used to precisely estimate the genetic diversity of different populations of *L. maculatus*.

Chi-square tests showed that the six microsatellite loci implied an extremely significant deviation from HWE. Meanwhile, the heterozygote deficiency which can lead to the departure from HWE was also indicated by the positive values of Fis for each locus (Table 1). The departure from HWE could be induced by many factors, such as genetic drift, small population size, null alleles, genetic mutation, non-random mating and Wahlund effect^6,27^. In the present study, the genetic drift can be excluded as the overall large Nm value (Nm > 1) among populations, the sample size of each population is also bigger than former studies^1,7–13^. Therefore, it might result from the high mutation rate of the specific nucleotide in the sequence targeted by the primer, which can lead to failure of PCR amplification and the detection of null alleles in this study (Table 1)^28^. Similar results could also be found in earlier studies^15,23,24,29–31^, which indicates this was a common phenomenon when microsatellite markers are used in population genetic researches. Meanwhile, the Wahlund effect cannot be ruled out as overall Fst (0.1196) is close to mean Fis (0.1325) (Table 1)^6^. In addition, as shown in Fig. 2, stratification within population leading to the existence of subpopulations could also contribute to the deviation from HWE in this study^23^.

### Population genetic diversity

In our study, the overall genetic characteristics identified were similar to that in earlier researches^1,15,23,29^, and higher than that assessed by mtDNA markers^14^. It is suggested that these 11 microsatellite loci were sufficient to evaluate the genetic information in the present study. In addition, the genetic characteristics of BH population sampled from the Bohai Sea were higher than that reported by Shao et al^23^. The higher value of genetic parameters in this study might result from the larger number of *L. maculatus* individuals used. As the allele number and the mutation rate at each polymorphic locus are positively correlated with to the sample size^29^. Furthermore, owing to the high resolution of capillary electrophoresis labeled by fluorescent markers, the genetic diversity parameters were obtained with higher value and accuracy compared with traditional methods^31^.

According to the PIC values, all eight *L. maculatus* populations showed a high genetic diversity (PIC > 0.5)^26^ and could be arranged in the following order: GY > BH > RD > FC > CM > QD > DT > LY. This is consistent with the notion that the genetic resource from the northern areas of China is better than that from the southern areas^12,15,32,33^. However, the genetic diversity of QD population from the northern area was much lower in the present study. These is because that *L. maculatus* individuals from northern coastal regions of China are widely acknowledged to have better environmental adaptability and growth performance^1,10,32,33^. Therefore, large number of wild individuals were captured and transported to southern aquaculture areas of China from the 1990s^1^. The situation was particularly serious in QD, due to its convenient geographic traffic environment. Correspondingly, the population structure of this fish in Shandong Peninsula changed from 2000 to 2006^1^. In contrast, the other northern populations such as GY, BH and RD populations showed a better genetic conservation. For the *L. maculatus* populations clustered to southern group, FC population showed the highest genetic variability, followed by CM population. It is easier to understand the relatively higher genetic diversity of CM population, because it is located in estuary area of Yangtze River. These coastal waters were considered to be the natural breeding areas for aquatic animals due to the favorable environmental conditions and sufficient food supply^31^. The frequent gene exchange with other locations as indicated by the high Nm and low Fis values also played an important part (Table 3). As for FC population, its particularly high genetic diversity might derive from the strong genetic conservation due to the geographic isolation formed by Leizhou Peninsula^13,14^, and the supplement of germplasm resources from the northern region, which can be supported by its close genetic relationship with QD population.

### Genetic differentiation and genetic structure

In our study, the differentiations among eight populations all reached a significant level (*P* < 0.01). However, in a previous genetic analysis of five *L. maculatus* populations based on mitochondrial COI gene, four of ten pairwise comparisons indicated insignificant genetic differentiation^14^. This is because mitochondrial DNA markers are maternal inheritance and can be easily influenced by selective pressure. They are not sensitive enough when used to analyze the genetic structure and gene flow of populations located in a small geographical area. In contrast, microsatellite markers used in this study can identify the weak genetic differentiation due to serveral advantages, such as high polymorphism, co-dominant inheritance, wide distribution, abundance and rapid evolutionary rate^34^.

Eight *L. maculatus* populations in this study could be divided into two groups based on Fst value. QD, FC, DT, LY and CM populations clustered as one group, while GY, RD and BH populations formed the other group. Overall, the genetic differentiation was found at a low or medium level within each group, whereas a medium or high level was observed between them^35^. These results provide new evidence for the conclusion that the *L. maculatus* populations along China coastal regions has experienced significant genetic divergence, and differentiated into the northern and southern groups^1,12–14^. It can also be verified by the results of genetic distance, genetic identity, UPGMA tree, STRUCTURE genetic cluster analysis and DAPC in our study (Table 4, Figs. 1, 2 and 3). As the gene flow among eight population were strong enough (Nm > 1) to prevent the genetic differentiation resulted from genetic drift^36^. The geographic segregation, ocean currents and habit differences such as the lower water temperature in the northern area which can limit the dispersal capacity of fish, might result in the divergence of those two groups. It's worth noting that QD population was assigned to the southern group genetically, although it belongs to the northern group geographically. A similar result was also reported in previous study^14^. As mentioned above, the large scale of anthropogenic transportation of wild *L. maculatus* individuals from QD to the southern aquaculture areas of China in history should be dominant reason^1^. It can be proven by the highest value of Nm indicating the sufficient gene exchange between QD and FC populations (Table 3). Consequently, QD population showed a much closer genetic relationship with and similar genetic component to the southern populations. With regard to the other southern populations including DT, LY and CM, the convenient gene exchange between them should account for the clustering and their similar genetic component, as there was no obvious geographic barrier among them.

When K value equaled to 4, RD population was separated from BH and GY populations. This separation might result from the following reasons: Firstly, the lower water temperature in the northern regions of China coastal regions limited the migration capacity of *L. maculatus*. Secondly, the geographic barrier formed by Shandong Peninsula might separate BH population from GY and RD populations. Thirdly, the invisible ocean current might result in the difference in genetic structure between RD and GY populations^37^, as their geographical distance is relatively small and no obvious barriers exists between these two locations. According to the STRUCTURE analysis, RD population showed nearly no gene mixture with other populations and exhibited an unique genetic structure, accounting for the high genetic differentiation between RD and other populations (Table 3). Moreover, a high genetic diversity was also detected in RD population. All these results suggested the genetic resource of wild *L. maculatus* in this region was well conserved. It's worth noting that, although the genetic diversity was higher in the northern group, their larger overall Fis value especially in RD population, indicated a possibility of future population depression, as the lower water temperature has limited their gene exchange with other populations (Table 3). Therefore, the protection of breeding group in these populations and the gene exchange with other populations should be reinforced.

### Demographic bottleneck

According to bottleneck analysis, these *L. maculatus* populations might have experienced a recently consecutive genetic bottleneck. As for short time bottleneck events, they can only influence the abundance of alleles but not their frequency. In contrast, continuous bottleneck effects can both result in the change of genetic variants and decline of genetic diversity^38–40^. Correspondingly, QD, FC, LY and CM populations in our study showed a lower genetic diversity compared with the other four populations. In previous studies, Weihai, Beihai, as well as QD *L. maculatus* populations were confirmed to have encountered bottlenecks^1,14^. It was also reported that all three populations of *L. japonicus* in Korea had experienced bottleneck events, because the overfishing in history and degradation of the environment has led to a decline in the sea bass population^41^. The *L. maculatus* populations in China were also under a similar situation. In order to meet the increasing market demands, a large number of wild fish fries were captured and utilized in the artificial culture activities of *L. maculatus* in history. As a result, there was a long-term overfishing pressure for the germplasm resources of this species, and ecological environment of its habitats was also destroyed by the fast-growing aquaculture industry. These changes in the recent decades may contribute to the bottlenecks observed in our study. In our previous study, the *L. maculatus* populations in China was suggested to have experienced potential population expansion events after a period of small effective population size^14^. These results emphasized the importance of protecting in the germplasm resources of *L. maculatus* and its habitats.

As the solution, artificial breeding of the seedlings of *L. maculatus* can mitigate the overfishing pressure of wild fries, and the protection of marine environment and strict management of closed fishing seasons will aid in restoration of germplasm resources. Furthermore, the germplasm bank of wild *L. maculatus* from different areas of China should be well constructed.

## Materials and methods

### Ethics declaration

All sampling fish were not endangered or protected species. In China, catching wild *L. maculatus* from sea waters does not require specific permits. Our study was approved by the ECSFRI (East China Sea Fisheries Research Institute, China Academy of Fisheries Science, Shanghai, China), and the study was carried out in compliance with the ARRIVE guidelines. All experiments were performed according to national law and guidelines of the animal care and use policies set by ECSFRI.

### Samples collection and DNA extraction

**Figure 4:**
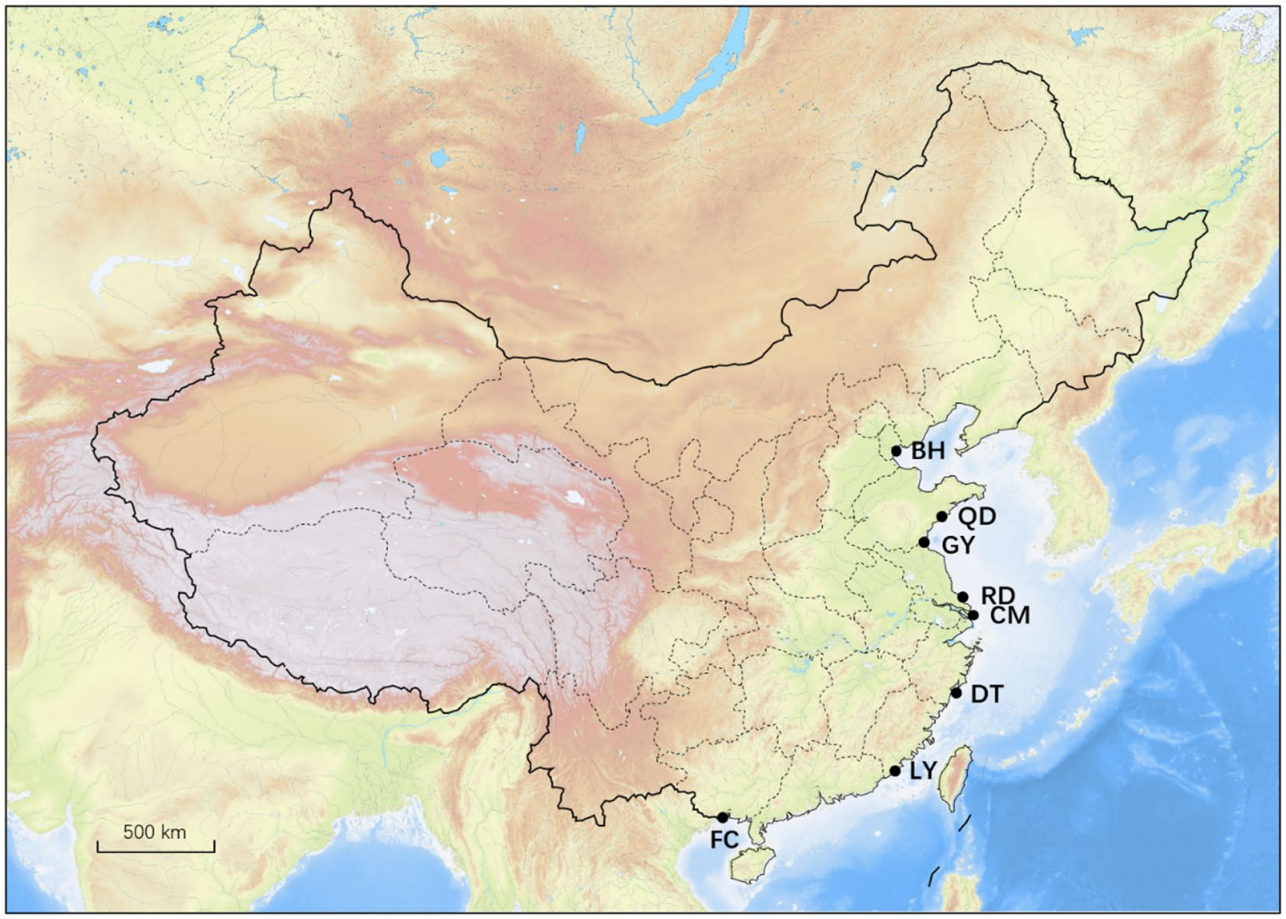
Geographical locations of the eight *L. maculatus* populations (generated by software BIGEMAP version 29.1.4.0, URL link: http://www.bigemap.com/). *BH* Binhai, *QD* Qingdao, *GY* Ganyu, *RD* Rudong, *CM* Chongming, *DT* Dongtou, *LY* Lieyu, *FC* Fangcheng.

A total of 294 wild *L. maculatus* individuals were collected along the coastal regions of China, including Binhai of Tianjin City (BH, N = 38), Qingdao of Shangdong Province (QD, N = 46), Ganyu, Rudong of Jiangsu Province (GY, N = 28, RD, N = 34), Chongming of Shanghai City (CM, N = 43), Dongtou of Zhejiang Province (DT, N = 25), Lieyu of Fujian Province (LY, N = 40) and Fangcheng of Guangxi Province (FC, N = 40) (Fig. 4). The sample size of each population in this study is similar or larger than that in previous related researches^1,15,23^, which is enough to provide scientific results. Dorsal muscle was sampled from each fish and genomic DNA was extracted using TIANamp Marine Animals Genomic DNA Extraction Kit (TIANGEN, DP324-03, China) according to manufacture protocols. The extracted DNAs were stored at − 20 °C until use.

### Microsatellite amplification

In this study, 11 specific microsatellite loci for *L. maculatus* as previously described were amplified after test^23^. The information of 11 microsatellite primers used in the present study was listed in Table 7. The fluorescent primers were synthesized with the 5' end of each forward primer labeled with a FAM or Hex fluorescent tag. PCR amplification was performed in a 20 µl solution consisting of 0.5 unit of Taq polymerase, 2.0 µl 10 × buffer, 0.5 µl dNTP (50 mM each), 1.0 µl each primer (10 µM), and 1 µl genomic DNA (50 ng/µl). Then ddH_2_O was added to the PCR mixture to make a final volume of 20 µl. PCR amplifications were conducted under a PCR touchdown protocol: initial denaturation at 95 °C for 5 min, followed by 10 cycles of 95 °C for 30 s, 62–52 °C for 30 s (reducing 1 °C for each cycle) and 72 °C for 30 s of extension, 25 cycles of denaturation at 95 °C for 30 s, alignment of 52 °C for 30 s, 72 °C for 30 s of extension, followed by a final extension step of 72 °C for 7 min. After that, the approximate size and concentration of PCR products were analyzed using agarose gel electrophoresis, PCR products were mixed according to non-overlapping fragment size and fluorescence marker (FAM or Hex), and then all samples were detected by capillary electrophoresis in an Applied Biosystem 3730XL DNA Analyzer sequencer, using LIZ 500 ladder as reference.

**Table 7:** Characterization of *L. maculatus* microsatellite primers.

Locus name	Primer sequences (5′–3′)	Repeat motif	Temperature (°C)	Allele size (bp)
Lama 04	F: TTGTTGTAAGTAGTGGTGGGAAT	(AC)9	54	172–176
	R: AAAAATGAAGGAGGACAGAATGA			
Lama 10	F: CAGACACACCCGAAAGAAAGT	(CA)9	54	224–230
	R: CTGAAGTTACCTGTCTCAAGCA			
Lama 21	F: TCCAGGTCTGTTTTCTGTTTC	(AC)13	54	242–260
	R: TTTTCTCGGATTATCTGTCTCA			
Lama 23	F: AACTGACGGAGATGATACGGT	(GT)15	54	148–154
	R: GCTGAAGAAGAGGCAGGTGT			
Lama 24	F: TCCCAAATCGTCTTGTCGGC	(GT)8	54	160–170
	R: CACACGCTGTTCACATTCTGCA			
Lama 28	F: AATCACGCAGAAAGTGGAAA	(CA)10	54	154–160
	R: AGACAGATGGGACGCATAAAC			
Lama 31	F: CCAGGGGGCAGACAGGAGGT	(TG)13	54	216–248
	R: GCCCCATTCTTCCTCCAACCA			
Lama 32	F: GTGCTGGTGCCTAAACGAACG	(CA)15	54	200–212
	R: TTTCCTGTGCTGCCTGGTGA			
Lama 36	F: CTAAAGGACCACAAGATACACG	(AC)13	54	276–280
	R: ACTCAGGCTCAAACCAGACA			
Lama 38	F: ACAAAACTCATCCATCAAGCAG	(GT)11	54	204–210
	R: AGTGTCCACGGAGACGGTAA			
Lama 44	F: GGGCAGTAATTGGTGAGGGA	(GT)16	54	148–160
	R: TCTTCAGGGCAAAAGGTGGT			

### Data analysis

The accurate product size and genotypes of all samples were analyzed using the software GeneMapper, Ver 3.0 (ABI, USA). All data obtained was imported into Microsoft Excel for further analysis. The presence of PCR errors such as large allele dropout, null alleles were investigated by the software MICOCHECKER^6,42^, allele frequencies (P_N_) for each locus and population were calculated by the EM algorithm (expectation maximization algorithm) using FreeNA^6,43^ The specific genetic diversity indices including observed number of alleles (Na), expected number of alleles (Ne), observed heterozygosity (Ho), expected heterozygosity (He), Shannon Wiener index (I), inbreeding coefficient (Fis), total in-coefficient of population (Fit) were calculated using POPGEN version 3.2^44^. Deviations from Hardy–Weinberg equilibrium (HWE) were tested using ARLEQUIN version 3.11, by using the Monte Carlo Markov Chain Method^45^. Polymorphic information content (PIC) was analyzed by PIC_CALV software version 0.6^46^. Nei's genetic distance (Ds) and gene flow (Nm) among different *L. maculatus* populations were computed by POPGEN version 3.2^44^. An UPGMA phylogenetic tree was constructed by MEGA software 5.0 version, following the Kimura-2-parameter (K2P) distance model based on the Ds values^47,48^. ARLEQUIN (version 3.11) was used to calculate the genetic differentiation coefficient values (Fst) and perform the analysis of molecular variance (AMOVA)^45^. The significance of AMOVA components was analyzed by 1000 permutations. Demographic bottleneck for each of eight *L. maculatus* populations was analyzed using BOTTLENECK version 3.4, based on Infinite Allele Model (IAM), Two-phased Model of Mutation (TPM) and Step-wise Mutation Model (SMM). The significance of difference excess heterozygosity was evaluated by Sign Test and Wilcoxon Sign-rank Test^49^. STRUCTURE (version 2.3) was employed to carry out a Bayesian clustering analysis. K value was set from 2 to 4, with a burn in period of 50,000 and a run length of 50,000. Five replicates were used for each K value, while the default values were set for the rest of the parameters. The optimum number of clusters among eight populations was also calculated by STRUCTURE software (version 2.3), which could be used to assess the theoretical population number based on the genetic structure of the eight *L. maculatus* populations^50^. Finally, discriminant analysis of principal component (DAPC) was performed by the ADEGENET in R (version 4.0.2)^51^.

## Data Availability

The datasets generated during and/or analyzed during the current study are available from the corresponding author on reasonable request.
